# The need for improved cognitive, hearing and vision assessments for older people with cognitive impairment: a qualitative study

**DOI:** 10.1186/s12877-019-1336-3

**Published:** 2019-12-03

**Authors:** Lucas Wolski, Iracema Leroi, Jemma Regan, Piers Dawes, Anna Pavlina Charalambous, Chryssoula Thodi, Juliana Prokopiou, Roxane Villeneuve, Catherine Helmer, Abebaw Mengistu Yohannes, Ines Himmelsbach

**Affiliations:** 1 Institute for Applied Sciences, Catholic University of Applied Sciences Freiburg, Freiburg im Breisgau, Germany; 2 Global Brain Health Institute, School of Medicine, Trinity College Dublin, Dublin, Ireland; 3 Devon Partnership NHS Trust, Wonford House, Exeter, UK; 4 The Department of Linguistics, Macquarie University, Sydney, Australia; 5 Manchester Centre for Health Psychology, School of Psychological Sciences, Univesity of Manchester, Manchester, UK; 6 Department of Health Sciences, European University of Cyprus, Nicosia, Cyprus; 7 University of Cyprus, Nicosia, Cyprus; 8 Université de Bordeaux, Bordeaux, France; 9 Department of Physical Therapy, Azusa Pacific University, Azusa, California USA

**Keywords:** Assessment tools, Dementia, Comorbidity, Visual impairment, Hearing loss, Qualitative research

## Abstract

**Background:**

Hearing and vision (sensory) impairments are highly prevalent in people with dementia (PwD) and exacerbate the impact of living with dementia. Assessment of sensory or cognitive function may be difficult if people have concurrent dual or triple impairments. Most standard cognitive assessment tests are heavily dependent on having intact hearing and vision, and impairments in these domains may render the assessments unreliable or even invalid. Likewise, dementia may impede on the accurate reporting of symptoms that is required for most hearing and vision assessments. Thus, there is an urgent need for hearing, vision and cognitive assessment strategies to be adapted to ensure that appropriate management and support can be provided.

**Objective:**

To explore the perspectives of PwD and the care partners regarding the need for accurate hearing, vision and cognitive assessments.

**Methods:**

We conducted focus groups and semi-structured interviews regarding the clinical assessment for cognitive, hearing and visual impairment. Participants (*n* = 18) were older adults with mild to moderate dementia and a sensory impairment as well as their care partners (e.g. a family member) (*n* = 15) at three European sites. The qualitative material was analysed according to Mayring’s summative content analysis approach.

**Results:**

Participants reported that hearing, vision and cognitive assessments were not appropriate to the complex needs of PwD and sensory comorbidity and that challenges in communication with professionals and conveying unmet needs and concerns by PwD were common in all three types of clinical assessments. They felt that information about and guidance regarding support for the condition was not adequate in the assessments and that information sharing among the professionals regarding the concurrent problems was limited. Professionals were reported as being concerned only with problems related to their own discipline and had limited regard for problems in other domains which might impact on their own assessments.

**Conclusions:**

The optimal assessment and support for PwD with multiple impairments, more comprehensive, yet easy to understand, information regarding these linked to conditions and corrective device use is needed. Communication among health care professionals relevant to hearing, vision and cognition needs to be improved.

## Background

The impact of aging due to increased longevity has become a health and social priority in Europe. With aging, cognitive decline and hearing and vision deficits emerge, often coincidentally, and the prevalence rate of all three types of impairments is rising [2, 37, 46]. Over 90% of older adults with cognitive impairment have hearing loss [19], and nearly one third of people with dementia (PwD) have vision impairment [5]; the proportion of older people with triple impairments is also increasing. In spite of this high rate of comorbidity, hearing and vision impairments continue to be under-recognised, under-diagnosed and under-treated ([1, 5, 9, 16]; Leroi et al., 2018, [55]).

Hearing and visual impairments may be accompanied by a decrease in cognitive performance [42, 58]. Comorbidity may worsen a range of dementia-related outcomes, including quality of life, and increase challenging behaviours such as agitation, aggression and depression [9, 24, 29, 38]. Older adults with dual and triple impairments may become more isolated from family interactions, participate less in social activities and hobbies, and become marginalised within the community (Lawrence et al., 2008; McKeefry et al.; 2010; Elliott et al., 2009; Lupsakko et al., 2002; McDonnall et al., 2009). Furthermore, caregiver burnout and physical exhaustion may be amplified due to greater dependency for self-care and other activities of daily living and communication barriers (Lawrence et al., 2008; Palmer et al., 1999). Recognition and management of sensory problems in PwD is thus a key aspect of improving outcomes for PwD.

There are several challenges in undertaking accurate cognitive, sensory and functional assessments in PwD. The first challenge is being able to distinguish whether an individual’s functional problems are due to their sensory or cognitive impairments, or both [5, 19]. An accurate assessment, considering all three domains, is key. However, standard clinical hearing and vision assessments may not be accurate since cognitive impairment may render a person less aware of their ongoing hearing or vision problems or less able to verbalise them appropriately. Moreover, many health care professionals tend to focus only on their own domain (e.g. vision, cognitive, or hearing impairment) and may pay less attention to comorbid problems that often present in older individuals [54]. There may also be limited, if any, communication among colleagues across the different disciplines or domains (Leroi et al., 2018). Moreover, the sensory assessment process itself may be affected by other factors commonly associated with dementia, such as depression, hallucinations, agitation, and anxiety that may add to the challenges in obtaining an accurate diagnosis in individuals with dementia. Other factors to consider pertain to the capabilities, knowledge and experience of the examining clinician, as well as their familiarity with the concurrent conditions and how they might affect the assessment process [6, 26]. Finally, aspects of the testing environment also need to be considered. For example, loud noises, distractors, and a stressful environment will have a greater impact on an individual with dementia compared to someone with intact cognition [25].

Importantly, from the cognitive testing perspective, for all the three domains (i.e. cognition, hearing and vision), distinguishing cognitive impairment from vision or hearing impairments and vice versa may be hampered by the lack of appropriately adapted cognitive screening tools for older people with dual or triple impairments [53]. Cognitive screening tests are generally very dependent on vision- and hearing-based test items, and clinicians in different disciplines have often adapted these in an ad hoc manner that may involve substituting or omitting vision- or hearing-based items completely [53]. This risks invalidating the test. Dual or triple impairments pose challenges to finding suitable approaches to optimally assessment. The question remains about how to obviate the obstacles presented in each type of assessment (i.e. vision, hearing or cognition), and how to develop the most appropriate treatment and care pathways for individuals once these combined problems have been identified and diagnosed.

The primary aim of the EU-funded SENSE-Cog research programme (www.sense-cog.eu) is to explore these and related issues in more depth. This will provide a basis on which to develop a support care intervention for PwD with concurrent hearing and/or vision impairments [34]. Here, we report one part of the SENSE-Cog programme that aims to explore the challenges in undertaking visual, hearing and cognitive assessments for people with dual or triple impairments, from the perspective of people with ‘lived experience’ of the condition(s) and their care partners in a different national and cultural settings in Europe. To do this, we collected qualitative data to explore the experiences of older adults with dementia regarding living with a concurrent sensory and cognitive impairment and their care partners. The findings from this study have informed a program of adaptation of assessments tools for people with dual or triple impairment (i.e. [10]), as well as clinical practice guidelines for clinicians and care workers across the three domains [35, 36].

## Methods

### Setting and design

This was a qualitative study using focus groups (FG) [30] and semi-structured interviews (SSI) in three European cities: Manchester, England, Bordeaux, France and Nicosia, Cyprus. All participants had recently undergone clinical assessments for cognitive, hearing and/or vision functioning, and the interviews were conducted between October 2016 and January 2017 at each of the three study sites. Ethical approval for all sites was granted as *per* local requirements and all research was conducted according to standards set by World Medical Association’s Declaration of Helsinki and the study conduct was guided by the principles of Good Clinical Practice [20].

### Training of focus group and semi-structured interview facilitators

Doctoral and postdoctoral Researchers from each site received extensive group and individualized training on qualitative methodology in a clinical context. The content of the training sessions included: (1) interviewing skills with different participants, particularly considering issues such as cognitive and sensory vulnerabilities; (2) transcription of material in a proscribed manner; managing qualitative data with QDA Software (MAXQDA 1987–2017 [41]); and (3) assessment of capacity to consent to participation, with a focus on people who might lack capacity.

### Participants

Participants were recruited over a three-month period from memory, audiology and vision rehabilitation clinics (community and university-based) at each of the three sites. We undertook a purposive selection of participants from consecutive clinic attendees (between September 2016 and November 2017) to ensure a representative range of socio-demographic characteristic (gender, age, education level) and stage of dementia (mild to moderate). Inclusion criteria for the participants with dementia were: age over 60 years, having an established diagnosis of dementia (due to Alzheimer’s dementia, vascular dementia or a mixed type), in the mild-to-moderate stage, able to speak and understand the local language of the site, and have self-reported hearing or a visual impairment severe enough to interfere with their activities of daily living. Older adults having an unstable, acute or current psychiatric or physical condition severe enough to prevent them from participating in the study, as determined by the investigator, as well as complete blindness or severe visual impairment or deafness (profound hearing loss), were excluded from the study. Inclusion criteria for care partners were: age at least 16 years; being the primary person responsible for unpaid support/care for the PwD at least 4 h per week in the community; willing to be a co-participant in the study; and able to speak and understand the local language of the site.

The sample size of the study (*n* = 18 PwD and *n* = 15 care partners) is appropriate to cover the information that is needed to identify relevant codes and themes in the given sample [21]. Since a long time there is discussions how many participants are needed to reach data saturation. In the qualitative context, data saturation is not about the numbers per se, but about the depth of the data [17].

### Interview topic guide

#### Development of the guide

FGs and SSIs were conducted using an interview topic guide, which was iteratively developed using data from different sources, over a period of 6 months prior to the study start of the interviews. The interview was developed for the SENSE-Cog FGs and SSIs, and has not been published elsewhere before. Firstly, the gaps and potential solutions regarding the assessment and support for PwD with sensory impairment were elicited through a multidisciplinary, international Expert Reference Group (The SENSE-Cog ERG), held over a two-day period in Athens, Greece in 2016 (Leroi et al. 2018). Secondly, findings from the relevant vision, hearing and cognitive impairment literature were considered (see overview in [11]), including specific data from other complex interventions for sensory impairment [8, 27, 33]. The information gleaned from these steps informed a prototype list of questions that were then sent to a panel of trained SENSE-Cog research associates and site principal investigators for consultation. Feedback led to a working draft of the topic guide that was then sent to SENSE-Cog’s trained patient and public voice (PPV) Research User Group (RUG [49];) in each of the three sites (Manchester, Nicosia and Nice, France) for consultation, feedback and verification of translation accuracy. Key issues for the consultation were the duration and ease of administration, use of language, level of understanding and other key aspects related to delivery of the questionnaire. The final version of the topic guide was submitted for approval and applied in the FGs and SSIs leading to a working draft of the interview guide.

#### Content of the guide

The main foci of interest related to the experiences of participants when undergoing cognitive, hearing and/or vision clinical assessments in the recent past, including whether the assessments provided them with enough information, and, for care partners specifically, whether the assessment helped them to support the PwD. We were also interested in exploring participants’ (PwD and care partners’) perceptions, knowledge and understanding of the of the clinical assessment process (see Additional file 1 and Additional file 2). Additionally, issues related to the clinical assessment of cognition, hearing and vision problems in context of comorbidity were also elicited. This was considered to highlight the gap in knowledge, understanding and availability of appropriately validated clinical assessment tools available for PwD and concurrent sensory impairment (Leroi et al. 2018 [53];). The facilitators posed the questions in an open-ended manner, followed by probes to elicit more detailed information, particularly related to barriers and facilitators to effective and informative assessments.

### Procedure

The facilitators and support team for the PwD all had experience of working with older PwD and sensory impairment, and we aware of the objectives of the study and the research questions. The interviews followed the interview topic guide and were conducted according to Witzels [60] problem-centred interview methodology. The focus groups (FGs), opted for by all participants in Manchester (*n* = 8 PwD; *n* = 6 care partners) and some participants in Nicosia (*n* = 5 PwD; n = 5 care partners). Using the same interview topic guide as the FGs, home-based semi-structured interviews were conducted with a further group in Nicosia (*n* = 3 PwD; n = 3 care partners), and Bordeaux (n = 5 PwD; n = 5 care partners). The interviews lasted between 60 to 120 min, following the consent signing process. The facilitators asked participants to introduce themselves, and then explained the purpose of the FG’s/SSIs and explained the conduct of the procedures. Each PwD was supported by a study assistant experienced in working with older adults with dementia. If at any point a participant wanted to leave the room or be with their care partner, this was facilitated. This occurred on one occasion with a single participant at the Manchester site.

All interviews were recorded with a digital Dictaphone. The interviews were transcribed in full to include all spoken words and non-verbal utterances such as sighs and laughter. Recordings were listened to and transcribed by the Research Associates at each site (Manchester, Bordeaux and Nicosia). Afterwards they were sent to the researchers at the Catholic University of Freiburg (CUF) who are responsible for qualitative data management and lead qualitative analysis. CUF received all qualitative data from each site in their original language (except the Greek ones - those were translated beforehand into English language). Multi-language material was treated according to the recommendations by Haak et al. [22].

### Data analysis

The SSIs and FGs data were analysed according to Mayring’s [45] summative content analysis. As pointed out by Krippendorff [30], as well as Elo and Kyngäs [14], the use of content analysis is a good method for making replicable and valid inferences from data regarding context, with the aim of providing knowledge, new insights, a representation of facts, and a practical guide to action. The authors at CUF Freiburg (LW and IH) coded the transcripts independently by using Qda-software (MAXQDA 1987–2017) as a tool for qualitative data management. Once three interviews of each FG and SSI were coded, the coder met to discuss different sub-codes and codes as well as to compare their individual notes. The initial coding framework was grounded in the content of the data (inductive). Once all transcripts were coded, the codes were put into broader themes and associated sub-themes. The main themes identified for the current paper are based on summative content analysis, the theoretical background and the research question. The themes deal with the perception of the assessment process and reflect different participants’ perspectives.

## Results

### Participant characteristics

Most of the participants were older females. The mean (SD) age of male (*n* = 8) and female participants (*n* = 10) with dementia was 77.4 (10.1) years (Range: 54-97 years). Most of the participants lived either with their spouse (n = 10) or a close family member (e.g. daughter, niece) (*n* = 4). Care partners mean (SD) age was 67.3 (15.4) years (Range: 30-88 years). All PwD were in the mild-moderate stage of dementia, with at least 3 years duration of clinical symptoms, and required some support with activities of daily living. The specific type of dementia was not reported in nine PwD as access to clinical records was not always available. Of the remaining PwD, four had an Alzheimer’s dementia (AD), two had mild cognitive impairment (MCI) of the amnestic type, two had vascular dementia (VaD) and one person had a mixed type of Alzheimer’s dementia as well as a vascular dementia (AD+VaD). The presence of hearing and vision impairment was based on self-report, and all participants had one or both impairments severe enough to interfere with daily functional ability. A summary of participants’ demographic characteristics is provided in Table 1.

**Table 1:** Demographic characteristics of people with dementia and their care partners

Clinical Site	Participant’s ID	Type of dementia	Hearing (H) and/or vision (V) impairment	Care partners ID	Type of relationship	FG or SSI
MANCHESTER	AP01	NR	H + V	S01	Spouse	FG
(MAN)	AP02	NR	H	S02	Spouse	FG
	AP03	NR	V	S03	Spouse	FG
	AP04	NR	H	S04	Spouse	FG
	AP05	NR	H	–	–	FG
	AP06	NR	H	S06	Spouse	FG
	AP07	NR	H	S07	Spouse	FG
	AP08	NR	H	–	–	FG
BORDEAUX	BP01	NR	H + V	SB01	Daughter	SSI
(BDX)	BP02	AD	H	SB02	Spouse	SSI
NICOSIA	NP01	VaD	H + V	SN01	Daughter	SSI
(NIC)	NP02	MCI	V	SN02	Spouse	FG
	NP03	VD	H + V	SN03	Spouse	FG
	NP04	AD	V	SN04	Spouse	FG
	NP05	MCI	H	–	–	FG
	NP06	AD	NR	SN06	Caregiver	FG
	NP07	AD+VaD	V	SN07	Daughter	SSI
	NP08	AD	H + V	SN08	Niece	SSI

Key: *NR* not recorded; Types of dementia related to their diagnosis: *AD* Alzheimer’s dementia, *VaD* vascular dementia, *MCI* mild cognitive Impairment, *AD + VaD* mixed type, *FG* focus group, *SSI* semi-structured interview

The following results reflect the findings from the FGs and SSIs regarding the clinical assessment process in all three domains, the PwD and the care partners’ knowledge and understanding of their condition, as well as their perceptions and understanding of any sensory support aides (i.e. hearing aids, glasses) they may have been using. The findings support clinical and care recommendations. The qualitative material revealed the following themes: (1) hearing, vision and cognitive assessments were not appropriate to the complex needs of PwD and sensory comorbidity; (2) challenges in communication and conveying unmet needs and concerns by PwD towards care partners and professionals were common in the different domains; and (3) that information about and guidance regarding support for the condition was not adequate in the assessments.

### Clinical assessments for hearing, vision and cognitive impairment are not ‘fit-for-purpose’

A key aspect that emerged from the interviews was that PwD acknowledged that they had received information (verbal or written) regarding the process and outcome of their clinical assessments in the different domains, but, in spite of this, they acknowledged that they were still *unable to explain the nature or impact of their impairments*, as well as the clinical recommendations made by the professionals. The option of home-based assessments, generally considered to be a helpful service for older people, was met with conflicting responses. Some PwD felt that *home-based assessments were an invasion of their privacy*, and that the assessment, particularly if equipment is required, might not be as thorough as when performed in a dedicated clinic space.

AP01: I’d prefer to go to the opticians because they’ve got more technical equipment there to test your eyes, because I have bad eyes and I wouldn’t want somebody to come to the house and do it, I’d rather go there and get through that whole procedure. (FG MAN PwD:32–38)

Several PwD expressed that they felt highly vulnerable with an unknown person seeing them in their own home. Some reported that they had been victims of ‘door step’ scams and fraud in the recent past. Thus, *trust and security issues* were of paramount importance and appeared to be a key factor in influencing the views of PwD regarding home visits. This issue is particularly important for older people with cognitive and sensory deficits; their ability to easily appraise and interpret an unknown person’s motives may be more limited compare to cognitively healthy older people with intact sensory functioning. Throughout the interviews, a repeated theme, raised by all participants, was the importance of knowing their clinicians well, and *having the same clinician* in subsequent visits.

They disliked working with new clinicians or having to discuss their concerns with new professionals at each assessment or visit.

R: Do you think that your optician understands that?

AP03: Well you don’t see the same one each time, it’s a different one every time you go. It’s a it’s like um, it’s not that one, no, I can’t think what it’s called. It’s probably in that letter in me pocket.

R: So would you, do you think you would find it more useful or helpful if it was the same person each time?

AP03: Errr well yeah but you, it’s only once every 2 years so you forget who you going to anyway within 2 years wouldn’t you?

(FG MAN PwD:83–87)

The practical and operational aspects of clinical appointments was another issue raised by both PwD and care partners. PwD admitted that they often *could not remember to keep their appointments* and reported that few clinics or services had robust appointment reminding systems in place. This creates challenges as people living with multimorbidity often must attend many different clinical appointments in different locations. Thus, a practical, easy-to-use reminder system could significantly enhance the clinical assessment process for PwD and sensory impairment. Furthermore, confining all sensory and cognitive assessments to the same place, and, if possible, date, would enhance attendance, lessen anxiety and increase the utility of the assessments for all stakeholders.

Another issue raised by PwD and care partners was that they felt that professionals’ clinical interests and expertise were limited to single systems and thus, what the participants perceived as their *complex and inter-linking problems were incompletely addressed*. For example, by focussing exclusively on cognitive impairment, the impact of hearing and vision impairment on an individual’s ability to perceive and make sense of his world, is not considered. Some felt their cognitive problems were being used as an excuse by professionals to *neglect addressing their hearing and vision problems* more thoroughly. This view was supported by the fact that for some PwD, once cognitive enhancing medication was prescribed and a diagnosis of dementia given, further assessments for non-urgent health concerns were neglected, notably hearing and vision assessments.

Care partners further reported their relatives might *experience physical and psychological distress* during assessments due to lack of understanding, and that this might impact negatively on the outcomes or findings of the assessment.

They have also reported that the assessment outcomes may lead to distress, anxiety or upset in the PwD, particularly if diminished insight or inability to recognise their impairments was present.

SB02: De vision, oui, il refuse *[Regarding vision, yes, he refused].* On lui a fait des ordonnances *[We wrote him prescriptions]*, il les met à la poubelle *[that he put in the garbage].*Et puis l’audition *[And for the hearing]*, il entend très bien *[he hears very well],* même si ce n’est. pas vrai *[even if it’s not true].*

(SSI BDX CG:2–2)

Thus, there is a strong risk that the clinical assessment process in any of the three domains may be misunderstood by PwD. Once again, this underscores the need for professionals to clearly explain the purpose of the assessment, the procedure to be undertaken and to appropriately manage expectations. On the positive side, care partners reported that if another support professional, such as an occupational therapist, was involved in the assessment process and was able to address psychological issues and support uptake of newly prescribed devices, such as hearing aids, outcomes for PwD were more positive and adherence to interventions and acceptance of the diagnosis was enhanced.

### Challenges in communication and information sharing in hearing, vision and cognitive assessments

The theme of ‘*challenges in communication in clinical settings’* was raised by several participants at several different points in the interviews. They reported having *difficulties making their specific unmet needs and concerns fully understood* by the professionals involved in their care, particularly in relation to sensory-cognitive health. PwD reported that their own care partners, as well as the professionals in the different clinical domains, did not take the time to hear their concerns fully. Thus, there was a risk that misunderstandings regarding needs, concerns and clinical complaints might arise.

AP07: […] It [*my condition*] has taught me to listen to people. If they’re talking to me, you know, really listen to what they say, which I think some, some, I don’t say generalised but some people don’t listen to what you say to them. So if I want to know I’ve got to listen.

(FG MAN PwD:187–191)

PwD also reported that *they did not feel comfortable asking for more information* or further clarification. Furthermore, they were unable to report on the details of what had taken place in the assessments in any of the domains, particularly the memory clinic assessments. This suggests that the means of conveying information in clinical assessments is not ‘fit-for-purpose’ and alternative means are necessary. The following quote illustrates that the assessment was indeed correctly undertaken by the professionals, but the PwD did not feel confident enough to ask for further information. It is possible that patients, particularly those of an older generation and/or having less confidence due to sensory-cognitive impairment, may not feel empowered enough to raise queries in what they perceive as a hierarchical physician-patient consultation.

R: Do you believe that the assessments provided you with all the necessary information?

NP08: Yes yes

R: If you had any questions

NP08: No, he didn’t even ask me he just examined my eyes …

R:. You... if you...

NP08: I am not asking … I don’t.

(SSI NIC PwD:192-198)

Some care partners suggested that professionals were more prone to providing a practical solution such as a prescription for medication rather than focussing on the individual challenges or patient’s expressed needs. This may, in part, be prompted by the specific methods of medical remuneration in some health systems, such as in Cyprus, as illustrated by the following two quotes:

NP08: […] I mean we have these problems we can help in these ways let’s say. They write you a pill/prescription they say go away and don’t come, she didn’t hear this just once, she heard it many times from an orthopaedic, ophthalmologist and from […] (FG NIC CG:187–201)

R: So you believe that these results let’s say could have been presented in a more meaningful way.

NP08: Yes, it’d have been better if the doctor put some effort to approach the patient.

R: Hmm. Do you think that you had all the information that you needed through these assessments?

NP08: They were explaining only when I was asking […]. In the private sector, yes, I had all the information […] (SSI NIC CG:187–201)

Several PwD also reported that consultation and clinical *assessment time is often too short*, and verbal explanations of procedures are not given in a clear and stepwise manner, assuming to much prior knowledge or understanding. In some cases, clinicians resorted to the use of jargon, further obscuring the ability of the patients to fully grasp the procedures being undertaken. This concern extended to challenges regarding the explanations about the use and maintenance of new sensory devices, particularly hearing aids, which may be introduced during or immediately following the assessment procedure.

AP02: Nobody has ever told me how to clean the plastic in these wee things [*referring to hearing aids*]. You know what I do? I take that off there and I hang it in an egg cup of warm water.

And then I clean them out with a pin which I don’t presume does the plastic very much good, but it does keep them clear for me. (FG MAN PwD:58–59)

None of the PwD and their care partners were able to offer a solution to this challenge*.* Thus, professionals should take time to carefully discussion outcomes of the assessments and the clinical recommendations, particularly involving new procedures that patients may have to adopt.

### Assessments do not provide adequate information and explanations for care partners

The care partners from different sites reported that the *assessment did not contribute to improving their understanding* nor their ability to understand or cope with the manifestations of conditions in the PwD. Furthermore, *additional health information was not routinely offered* by the professionals in the different domains. Thus, care partners reported that they had to source further information regarding the cognitive and sensory conditions themselves. This is illustrated in the two quotes below:

R: so there wasn’t much of an explanation?

SN02: [...] yes more explanatory, this might have happened because there are different doctors and they are very busy …

R: neither for how to take care...

SN02: They have to inform us more

R: […] yes and to tell you and how to take care maybe...

All: Yes yes (in chorus)

R: about the changes, nobody told you.

SN02: They expect to learn from us.

(FG NIC CG:1088–1096)

S01: Well, we remember doing the test and at the end she said you got 27/35 or something.

R: So there’s no kind of meaning?

S01: No.

R: Just a score.

S01: I know what she got wrong, […] I know she can’t do it, she can’t. But I mean that’s it, I got nothing, I got nothing else. Just a score.

S04: The score, the score anyway can’t be meaningful, cos everyone’s different. S07: So it can’t be meaningful. (FG MAN CG:136–143)

This demonstrates that test results may need to be explained more comprehensively and appropriately contextualised in clear lay terms for both the PwD and their care partner.

It should also be noted that the experience of obtaining adequate information and explanations appeared to differ somewhat across the three health systems represented in the study (United Kingdom, France and Cyprus). Furthermore, in privatised systems, such as in Cyprus, in which patients pay for each consultation, there may be additional challenges in obtaining the necessary access to multidisciplinary care and health information [57]. In contrast, in socialised medical systems, such as in the United Kingdom, access to different professionals representing the different domains was more possible but tended to be fragmented and located in different health care organisations.

## Discussion

The qualitative explorations of this study revealed that clinical assessments for sensory and cognitive functioning in older adults do not adequately meet the needs of PwD with concurrent hearing and/or vision impairments for several reasons. These include a lack of appropriate information offered by professionals, inadequate information sharing among professionals about the PwDs complex needs, limited consultation time, lack of knowledge and skills in professionals regarding concurrent yet highly prevalent conditions which may impact on the primary assessment domain, and other operational aspects about how clinics and assessments are conducted. Another important finding was that PwD and their care partners do not feel empowered enough to obtain to necessary information about the condition of the PwD. These issues may leave the PwD and their care partner with unmet needs and queries and may even be associated with psychological distress or anxiety.

It was clear from the interviews that although all the PwD had undergone both sensory and cognitive assessments, their understanding of their condition was limited. This raises the opportunity for several recommendations regarding the conduct of hearing, vision and cognitive health assessments.

### Clinical assessments for hearing, vision and cognitive impairment are not ‘fit-for-purpose’

The qualitative findings revealed that it should be considered offering home-based assessments in all three domains if available and the person is home-bound or attending a clinic setting might not be possible. Also findings from Wong and Jacova [61] indicate that (in a population including older adults having no cognitive impairment) taking tests at home is well accepted by older adults.

But as to PwD their abilities to easily appraise and interpret an unknown person’s motives may be more limited compared to cognitively healthy older people with intact sensory functioning and thus may lead to distress or anxiety [23, 28]. Especially for patients with dementia, assessments via Telemedicine (e.g. using direct-to-home videoconferencing technology) might also be an appropriate solution [39]. For those having concerns regarding usability issues (e.g. handling a telecare device) [48] however, clinic-based appointments should still be an option to take an assessment. Apart from that educational aspects respectively common knowledge regarding sensory as well as cognitive impairment need to be strengthened. When talking to patients and their care partners about their experiences, during an assessment, a lot of them seem not to be able to understand and integrate the given information properly (e.g. most of all concerning the progression of the neurocognitive disorder as well as the ongoing assessment procedures) (Maslow & Fortinsky, 2017 [40]). The type of assessment doesn’t seem to make a difference here, whether it is a hearing, visual or cognitive assessment. Regarding the neuropsychological assessment a study by Westervelt et al. [59] showed, for example, that both the patients and the significant others had positive attitudes towards a feedback on the assessment. Besides, recommendations about the disease were perceived as helpful. As distinct from other studies regarding sensory impairment, PwD got to be involved in the SENSE-Cog FG sessions. Thus, some of the findings might be biased due to the ongoing neurocognitive disorder. But even the care partners were not able to reconstruct the patient’s assessment history properly. Hence, the assessment is moderated by several factors, such as expectations regarding the assessment, perceived relevance, evaluation length, provision of feedback, and its perceived usefulness [3]. From a qualitative point of view, it can be recommended, that health care professionals should spend more time on enlightening the care partner about the disease, the use of technical devices as well as the diagnostic process. This might contribute to enhance the patient’s (and care partners) satisfaction. Like findings from Donofrio et al. [13] could illustrate, a short written report for the care partner is perceived as especially helpful to provide further understanding. Moreover, after having given an explanation regarding an aspect of the assessment or the patients’ condition, it is important for professionals to carefully ask the patient to briefly re-state the information to ascertain their level of understanding.

Another point that was raised focused on the fact that PwD have trouble to remember to keep their appointments. Therefore it is important to offer robust, easy-to-use appointment reminder systems by clinical services in all the domains. Beyond that services should work together to combine sensory-cognitive assessments in single locations on the same date, if practicable. Probably this would reduce the chances of missed appointments, increase the chance that multimorbid sensory-cognitive problems will be detected, and foster better communication among the respective clinicians.

Like mentioned elsewhere, PwD and their care partners had the idea that arising problems with sensory and cognitive problems were only incompletely addressed by the health care professionals. Some of them felt that the health care professionals neglected addressing their hearing and vision problems adequately. Regular sensory assessments for PwD could remedy this and should be included in care plans and not cease following diagnosis of dementia. Besides it is recommended that clinicians in each of the three domains should have some knowledge, skill and practice regarding basic screening in the other domains. For example, a memory assessment clinician should undertake basic hearing and vision screening; a hearing or vision clinician should ask about cognitive and other sensory impairment and might even administer a simple cognitive screening tool (i.e. the GPCOG [7] or the 6CIT [18]).

### Challenges in communication and information sharing in hearing, vision and cognitive assessments

Throughout the interviews we see that communication has an impact on different aspects like the relationship between the care partner and PwD or the assessment situation itself. Except for sensory impairments Lin and Whitson [38] indicate, that aspects like different literacy levels, cultural and language barriers, cognitive status, and environmental factors can threaten good communication, too. Corrective aids like hearing aids, for example, improve the understanding between the care partner and the PwD and also improve the understanding regarding a consultation with a dementia or sensory health care professional. Apart from that, it is important to focus on different aspects of communication with older adults having several impairments. Good physician-patient communication is essential to high quality health care, from history taking, to knowledge transfer, to understanding of discharge instructions, to patient self- management. Considering the added burden on communication posed by sensory-cognitive multimorbidity, it is important for professionals in all the domains to carefully listen to patients’ concerns and make efforts to fully comprehend their needs, concerns and reported symptoms.

Feeding back the concerns to ‘sense check’ with the PwD is important also to encourage to patient-initiated questions. Likewise good communication is also linked to better emotional health outcomes, better quality of life and safer care [56]. Unfortunately a lot of clinicians suffer time to conduct a thorough assessment. Clinical assessments for PwD and sensory impairment requires a longer time for the appointment and more information, conveyed clearly, using different various of communication and avoiding jargon; clinicians should not assume prior knowledge or understanding; consider a dedicated ‘cognitive-sensory clinic’ slot to ensure the needs of older people with more complex needs can be met. When conveying new information or offering an intervention following the assessment, clinicians need to carefully support the new process.

### Provision of more information regarding different impairments

Even though there are more and more public institutions that provide information regarding the progress of age related impairments, there still seems to be a considerable gap in knowledge in care partners on those topics. Some of them seem to hope that the health care professionals provide them with relevant information, but it is not uncommon that the much of the non-physician care partners only remain with limited knowledge (Maslow & Fortinsky, 2017). So, there is a need that health care professionals interact more with the care partners upon a trusting relationship that includes all persons involved.

## Conclusions

There is a call for improvement in communication strategies regarding different aspects like understanding and comprehension. Besides it is necessary for the care partners to receive more Information on technical devices and the progress of a neurocognitive disorder. This also means a better inclusion of the care partner throughout the whole assessment process. It is recommended that specialists take more time on patients with multiple impairments and contribute to broaden the knowledge on multiple impairments.

### Limitations

There are certain limitations of our study which we must acknowledge. Firstly, we were unable to recruit participants directly following their different assessments, thus, particularly for PwD, they may not have had been able to remember all the details of the assessments they had received. Nonetheless, both PwD and care partners appeared able to express their views in detail and it is likely that the most salient features would have come to the fore.

Another issue was the difference in interview methods among the sites: one site had only FGs, one had only SSIs, and the third had a mix. However, the topic guide used for both approaches was the same, and we felt that by tailoring the approach to the needs of the participants was important to enhance the accuracy and quality of the information obtained. Finally, there are certain challenges inherent in undertaking qualitative work with PwD, particularly when asking them to self-report on issues about which they may have incomplete understanding. From the discussions, it was evident that not all PwD recognised the extent of their different sensory-cognitive impairments and the impact these problems might have had on their daily functional ability. Nonetheless, exploring the views of people with lived experience of complex conditions is vital for a more complete, and meaningful, understanding of how to address a particular issue [4, 50]. An important strength of our work is that this is, to our knowledge, the first study to elicit the views of PwD and their care partners regarding their experiences of being assessed for sensory-cognitive health issues. With the rising prevalence of these comorbid conditions in our increasingly aged society, it is vital to adopt the most appropriate clinical practices to ensure the best outcomes for older people with complex health needs.

## Supplementary information

**Additional file 1.** Assessment needs guideline - PwD. Interview guideline focusing on the perception and experience of different assessments in the PwD.

**Additional file 2.** Assessment needs guideline - Care Partner. Interview guideline focusing on the perception and experience of different assessments in the care partner.

## Data Availability

The datasets used and/or analysed during the current study are available from the corresponding author on reasonable request.

## Abbreviations

AD: Alzheimer’s dementia
BDX: Bordeaux
CUF: Catholic University of Freiburg
ERG: Expert reference group
EU: European Union
FGs: Focus groups
GCP: Good clinical practice
MAN: Manchester
MCI: Mild cognitive impairment
NIC: Nicosia
NR: Not recorded
PPV: Patient and public voice
PwD: People with dementia
QDA: Software for qualitative and mixed methods research
RUG: Research user group
SSIs: Semi-structured interviews
VaD: Vascular dementia
