# Supplementary material for: The need for improved cognitive, hearing and vision assessments for older people with cognitive impairment: a qualitative study
Source: BMC Geriatr. 2019 Dec 3;19:328. doi: 10.1186/s12877-019-1336-3 (PMC6889573; doi:10.1186/s12877-019-1336-3)
Supplement: Supplementary file 2 — Additional file 2. Assessment needs guideline - Care Partner. Interview guideline focusing on the perception and experience of different assessments in the care partner. [file 12877_2019_1336_MOESM2_ESM.docx]

**Additional File 2: FG/SSI - Assessment Needs Care Partner**

| *No.1* | *Question* |
| --- | --- |
| *1.* | *“Can you tell us how the person you care for has been assessed in the past for their hearing, vision or cognitive problems?”* |
| *2.* | *“How did the assessment(s) make them feel?”* |
| *3.* | *“How could the assessment be better for them?”* |
| *4.* | *“Have you identified any problems or difficulties with the assessment(s)?”* |
| *5.* | *“What did the results mean for you and the person that you care for? Could the results be made more meaningful?”* |
| *6.* | *“Did the assessment(s) provide you with all the information you needed?”* |
| *7.* | *“In light of your results how did it change your understanding of the issues faced by the person you care for?”* |
| *8.* | *“After being assessed, did the life of the person you care for improve in any way? How did your life change after the assessment(s)?”* |
| *9.* | *“Did the results of the assessment(s) change the way that you and the person you care for manage the condition(s)?”* |
| *10.* | *“Do you think the results of your assessment are an accurate reflection of the problems faced by the person you care for?”* |
| *11.* | *“How did the assessment results help you with the caring process?”* |
| *12.* | *“Is there anything else you would like to share with us, which we missed out with our questions?”* |
